# Fire Source Localization Based on Distributed Temperature Sensing by a Dual-Line Optical Fiber System

**DOI:** 10.3390/s16060829

**Published:** 2016-06-06

**Authors:** Miao Sun, Yuquan Tang, Shuang Yang, Jun Li, Markus W. Sigrist, Fengzhong Dong

**Affiliations:** 1 Anhui Provincial Key Laboratory of Photonic Devices and Materials, Anhui Institute of Optics and Fine Mechanics, Chinese Academy of Sciences, Hefei 230031, China; sunmiao712@163.com (M.S.); laserway@163.com (Y.T.); franky.aiofm@foxmail.com (S.Y.); ljsiom@aiofm.ac.cn (J.L.); 2 Hefei Institutes of Physical Science, University of Science and Technology of China, Hefei 230029, China; 3 ETH Zürich, Institute for Quantum Electronics, Otto-Stern-Weg 1, CH-8093 Zurich, Switzerland; sigristm@phys.ethz.ch

**Keywords:** dual-line optical fiber, fire source localization, optical fiber distributed temperature sensor

## Abstract

We propose a method for localizing a fire source using an optical fiber distributed temperature sensor system. A section of two parallel optical fibers employed as the sensing element is installed near the ceiling of a closed room in which the fire source is located. By measuring the temperature of hot air flows, the problem of three-dimensional fire source localization is transformed to two dimensions. The method of the source location is verified with experiments using burning alcohol as fire source, and it is demonstrated that the method represents a robust and reliable technique for localizing a fire source also for long sensing ranges.

## 1. Introduction

Fire detection techniques play an important role in various areas by providing assistance to reduce the economic and ecological damage as well as casualties [1,2]. To avoid the excessive fire damage, sensor networks composed with cameras, flammable gas detectors, and temperature gauges have been implemented [2,3,4,5,6]. Localizing the source is the main concern. Fire intensity and size during the fire also enable forecasting [7,8,9,10]. Richards *et al.* used a zone model and exhaustive search with a black and white video camera to detect, locate and size accidental fires [7,8]. Xia *et al.* applied a model of a dual-line gas sensor array to collect information on concentrations of gases emitted by the fire and to calculate the time delays of the signals received from various array elements [3]. However, the localization results are easily affected by any obstructions present in the area of the fire place.

Nowadays, various methods based on temperature measurements have been proposed to locate fires as the temperature change is perhaps the most obvious sign when a fire occurs. The main research direction is that sensor arrays with four detectors are used to locate the fire source based on the time-delay estimation algorithm [7]. One or two sensor arrays are adopted to calculate the distance between the fire source and the sensor array with far field algorithms [5,6], and near field algorithms [11]. The proposed methods yield good results as long as the fire source is close to the sensor array. However, the reliability of the localization method may suffer in the case of a remote fire. Moreover, a large number of the sensor arrays are needed when the fire has to be monitored in a huge space. Thus it is difficult to overcome the limitations such as high construction costs, difficulty in installing signal-circuits, and difficulty in reducing measurement time when taking into consideration the temperature measurement with discrete sensors.

An optical fiber distributed temperature sensor (DTS) system is a kind of real-time and continuous monitoring space temperature field sensing technology [12,13]. The temperature profile can be derived based on the Raman backscattered light and the optical time-domain reflectometry (OTDR). The earliest example of distributed sensing with OTDR we could find is attributable to Dakin *et al.* [14]. A conventional optical fiber was used as the sensing element and the distributed sensor achieved a good spatial resolution of 3 m with sensor lengths up to 1 km. Numerous studies have been performed with the DTS, and a sensing distance >10 km has been achieved based on Raman backscattering [15]. The DTS has been widely used in many areas due to its unique advantages, such as immunity to electromagnetic fields, easiness of installing wire, remote distributed measurement compared with the conventional temperature sensors [16,17,18,19,20]. With an optical fiber as the sensing element, the DTS can realize temperature monitoring with long-distance and wide range, which solves the deficiency of the sensor arrays.

In this study, a novel method of fire source localization based on the DTS system is presented. Two parallel sections of multimode fiber are adopted as the sensing fiber. Hot gases are produced when the fire occurs in a closed room. The hot gases rise and propagate in a circular shape along the ceiling of the room. By analyzing the temperature changes measured with the DTS system, the localization of the fire source is examined theoretically. The experimental results show that the proposed method provides a reliable fire source location for long sensing ranges.

## 2. Theoretical Analysis

### 2.1. Theory of the DTS System

In a DTS system two Raman scattered components (Stokes (S) Raman scattered light and anti-Stokes (AS) Raman scattered light) are generated when a short laser pulse is launched into the optical fiber. Contrary to the Stokes Raman scattered light, the intensity of the anti-Stokes Raman scattered light strongly depends on the local fiber temperature. For the temperature determination, the anti-Stokes Raman scattered light is regarded as the signal light, while the Stokes Raman scattered light is seen as the reference light. Thus the temperature (*T*) dependence of the intensity ratio F(*T*) can be expressed as follows [21]:

(1) $$F(T)=\frac{\varphi_{AS}(T)}{\varphi_{S}(T)}=\frac{K_{AS}}{K_{S}}\frac{\nu_{AS}^{4}}{\nu_{S}^{4}}\exp[-(h\Delta \nu /k_{B}T)]\exp[-(\alpha_{AS}-\alpha_{S})l]$$

where $\varphi_{AS}(T)$ and $\varphi_{S}(T)$ are the intensity of AS light and S light at temperature *T*, respectively, *K_AS_* and *K_S_* are coefficients concerning AS and S scattering cross-section, respectively, *ν_AS_* and *ν_S_* are the frequencies of AS light and S light, *h* is the Planck constant, ∆*ν* is the Raman shift in the transmission medium, and *k_B_* is the Boltzmann constant, α*_AS_* and α*_S_* are the attenuation coefficients for the incident AS and S light, respectively, *l* is the location along the fiber.

To calculate the absolute temperature, the reference fiber coil with the fiber length *l*_0_ is maintained at a known temperature *T*_0_. Then the final temperature *T* can be obtained as:

(2) $$\frac{1}{T}=\frac{1}{T_{0}}-\frac{k}{h\Delta \nu }\ln(\frac{F(T)}{F(T_{0})})$$

### 2.2. Theory of the Fire Source Localization

**Figure 1:**
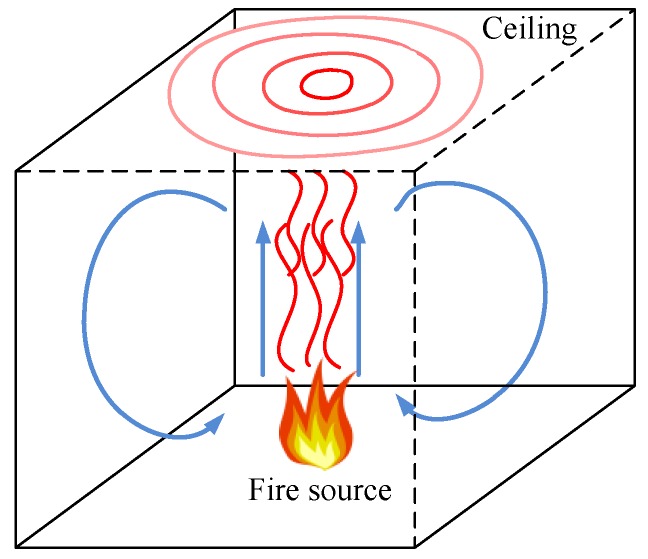
A fire in a closed room.

A near field algorithm using the DTS for the fire source localization is proposed in this paper. As shown in Figure 1, a fire breakout in a closed room will produce lots of hot gases. The hot gases rise to the ceiling of the closed room and then propagate in circular shapes across the plane of the ceiling. The flow of the hot gases will not be perfectly circular due to the impact of weak turbulences and the influence of the room walls. However, an almost circular gas flow is expected if a time average is taken. To simplify the problem, some assumptions are made. The ceiling of the closed room should be smooth with a low thermal conductivity. All walls are considered to have the same temperature. The fire is located below the ceiling and not directly at a wall. The flow velocity of the hot gases is considered constant under the ceiling in the early stage of the fire. Other currents such as air currents caused by an air conditioning system are neglected [5,11]. Under these assumptions the wavefront of the hot gases has a perfect circular shape. The fire source is situated at the zone underneath the center point of the circular shape and the coordinates of the center point correspond to the fire’s coordinates in the two dimensional plane. As a consequence, the problem of the three-dimensional fire source localization is essentially converted into a two-dimensional orientation determination of the center point of the flow of the hot gases. Obviously a more complex model would be needed if, e.g., the fire would be located near a wall or for non-symmetric configurations. However, as is demonstrated here, the proposed method enables a fast and efficient fire source location in many situations.

**Figure 2:**
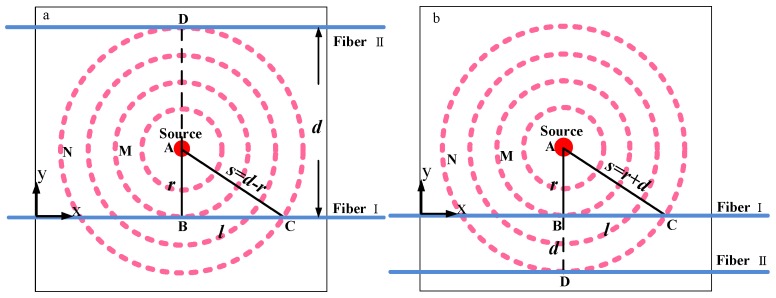
Fire source and the geometrical arrangement of the sensing fibers: (**a**) Fibers on opposite sides of fire source; (**b**) Both fibers on same side of fire source.

The two-dimensional fire source localization and geometrical arrangement of the optical fiber are shown in Figure 2, which is the top view of Figure 1, for two different configurations. The closed rings represent wavefronts of the hot gases, and each ring means a wavefront with identical temperature. Point A is the fire source location. The wavefronts M and N represent two different radii of the circular wavefront. Two sections of the optical fibers are placed parallel within the temperature field, crossing with different radii of wavefronts. The points B and D are the points of tangency between the fibers and the corresponding wavefronts. The assumption is made that the fire source is small enough so that it can be seen as a point fire source. Then the points with maximum temperature measured along the fibers are points B and D, respectively, and the smaller the radius, the higher the temperature. The position of the high temperature point can be determined by using OTDR, which is the abscissa of the fire source.

To determine the fire source location the ordinate of the fire source is required, which is the distance *r* between fiber I and the fire source. The measured temperature at point B is higher than the one at point D due to the smaller radius *r* of wavefront M compared to *S* of wavefront N. Obviously, there is one point C at fiber I at which the measured temperature is the same as at point D at fiber II, because points C and D belong to the same wavefront N. The distance between the two parallel fibers is *d* and *l* is the fiber length between point B and point C. Noticing the right triangle ABC the relationship can be expressed as:

(3) $$r^{2}+\;l^{2}=\;s^{2}$$

When the fire source is located between the two fibers (see Figure 2a), then:

(4) d−r=s

When the fire source is located on one side of the two fibers (see Figure 2b) we have:

(5) r+d=s

Thus the distance radius *r* can be expressed as:

(6) $$r=\left|\frac{l^{2}-d^{2}}{2d}\right|$$

With the above abscissa and the ordinate of the fire source, the localization of the fire source is defined.

## 3. Experimental Setup

**Figure 3:**
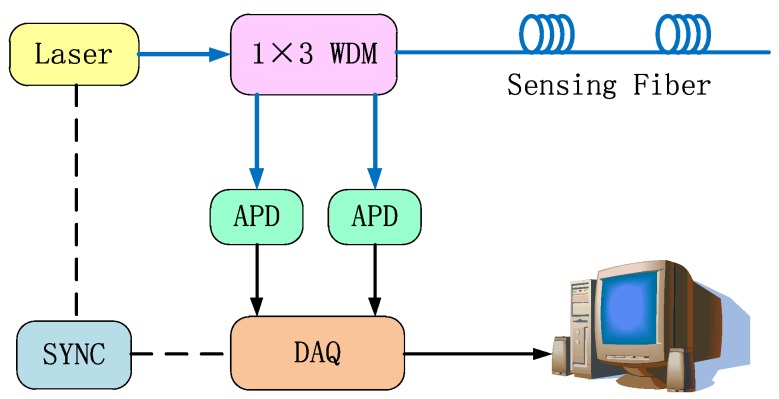
Schematic of the DTS system setup (WDM: wavelength division multiplexer; APD: InGaAs avalanche photodiode; DAQ: data acquisition).

The schematic drawing of the DTS system is presented in Figure 3. The short pulse of light from a pulsed fiber laser operating at 1550 nm with a maximum peak power of 30 W, 10 ns pulse width and 10 kHz repetition rate is injected into the optical sensing fiber through a 1 × 3 wavelength division multiplexer (WDM). Raman scattering occurs in the optical sensing fiber, and the backscattered Raman Stokes as well as the Raman anti-Stokes light passing through the WDM are injected into a high-performance InGaAs avalanche photodiode (APD). Using the APD, the Raman Stokes and anti-Stokes light are transformed into electrical signals. Then a high speed 100 MHz data acquisition card converts the analog signals to digital signals. Finally, the computer processes the digital signals and calculates the temperature data according to the theoretical analysis presented in Section 2.1.

**Figure 4:**
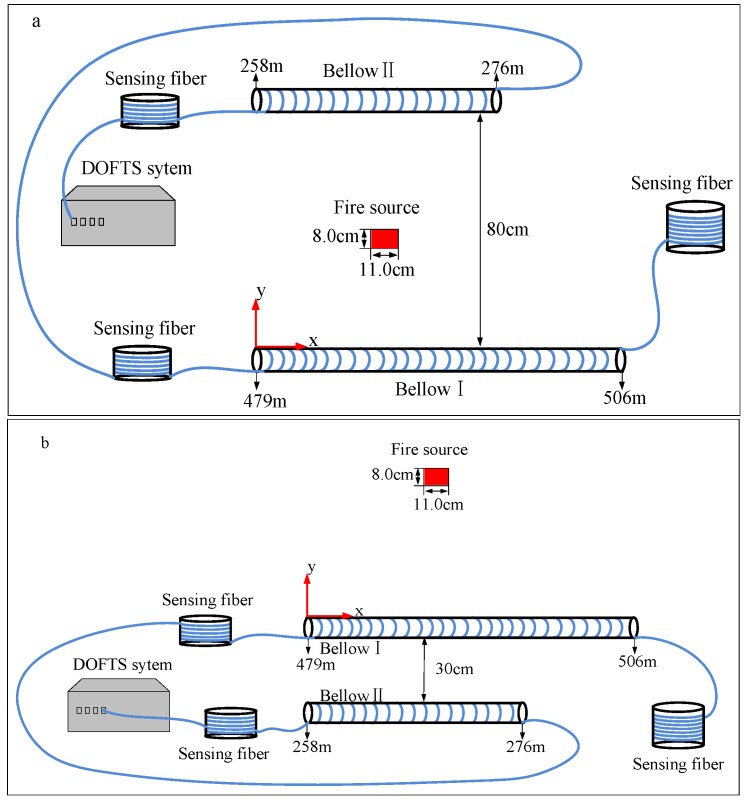
Experimental setup of the dual-line sensing fiber for fire source localization: (**a**) Fibers on opposite sides of fire source; (**b**) Both fibers on same side of fire source.

To verify the theoretical analysis fire source localization experiments were carried out in a laboratory in a simulated environment. The experimental setup is shown in Figure 4. A tray of alcohol (11.0 cm × 8.0 cm × 2.1 cm) placed on a table (2.4 m × 1.2 m × 0.8 m) is used as the fire source. In the closed laboratory room, the hot gases rise to the ceiling and propagate under the ceiling. Then the hot gases flow down at the walls and flow back to the fire source origin near floor level. Thus there is a hot flue gas layer with a thickness of 100–300 mm beneath the ceiling if the fire source is located far away from the walls. In order to simplify the experiments, a heat insulation roof (2.4 m × 1.2 m × 0.05 m) supported by four pillars is placed 1 m above the table. This roof is placed parallel to the table and acts as the “ceiling” as discussed above in Figure 1. The table, the four pillars and the ceiling form an open cabinet in the laboratory. If a fire occurs in the cabinet, the hot gases produced by the fire rise up to the heat insulation roof and propagate under the roof, forming a layer of the hot flue gases beneath the roof. Because the open cabinet is placed far away from the walls of the closed laboratory room, the hot gases propagating to the edge of the roof will continue to flow horizontally instead of flowing down when arriving at the room walls. There is no influence on the inner hot flue gas layer under the roof. As a result, the cabinet is employed to simulate a true situation of a fire with sensors placed far away from the edge of the roof. A standard multimode fiber with an attenuation of <0.3 dB/km at 1550 nm acts as the sensing fiber. The spatial resolution of the DTS system is limited to 2 m by the laser pulse width, APD bandwidth and the DAQ card sampling rate, which is much larger than the diameter of the fire source. An improvement of the spatial resolution is achieved by evenly winding the sensing fiber on bellows; the fiber length wound on every 100 cm bellow is 18 m. As a result, the spatial resolution of the DTS system is improved from 2 m to 11.11 cm. The bellows with the sensing fiber are placed just underneath the heat insulation roof with a distance of 50 mm, where the bellows are totally immersed in the hot flue gas layer. Thus the changes of the temperature can be measured with the DTS system in real-time.

## 4. Results and Discussion

**Figure 5:**
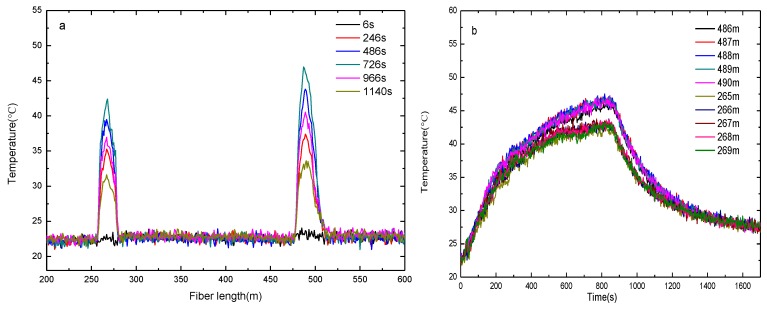
The measured temperature by the DTS system with fibers on opposite sides of fire source: (**a**) Temperature *versus* fiber length at different burning times; (**b**) Temperature *versus* the fire burning time for different fiber lengths.

**Figure 6:**
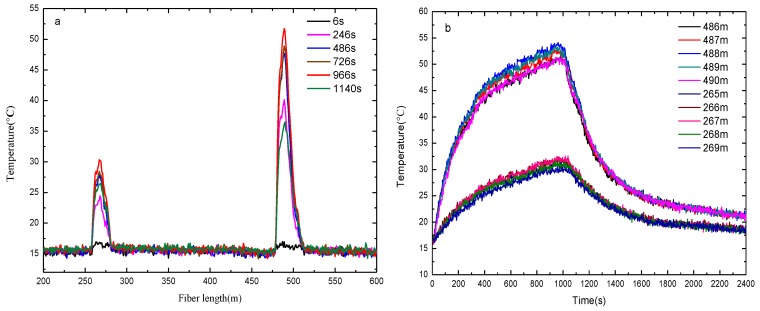
The measured temperature by the DTS system with fibers on same side of fire source: (**a**) Temperature *versus* fiber length at different burning times; (**b**) Temperature *versus* the fire burning time for different fiber lengths.

The two-dimensional plane coordinate system is shown in Figure 4. The left endpoint of bellow I acts as the origin of coordinate system and the direction of bellow I is the horizontal axis while the distance between the fire source and bellow I corresponds to the ordinate y of the fire source. The two parallel fibers are placed on opposite sides of the alcohol tray as shown in Figure 4a. The distance between the two parallel bellows is 80 cm and the coordinates of the alcohol tray center are (45 cm, 35 cm). On the contrary the two parallel fibers are placed but on the same side of the alcohol tray in Figure 4b. The distance is 30 cm and the coordinates of the alcohol tray center are (45.5 cm, 32 cm). Figure 5 and Figure 6 show the measured temperature by the DTS system when the alcohol is burning. The temperature measured by the fiber on bellow I is higher than the one recorded on bellow II, which indicates that the bellow I is nearer to the fire source. As shown in Figure 5a and Figure 6a, the length range of the sensing fiber winded on bellow I which records an increased temperature is 27 m (from 479 m to 506 m) and the one on the bellow II is 18 m (from 258 m to 276 m). The burning alcohol is a non-uniform heat source, and it cannot be seen as a point heat source. Moreover, some turbulence of the surrounding environment occurs in the process of measuring the temperature when the hot gases are rising to the heat insulation roof. Based on the above reasons, the position of the maximum temperature along the bellows is slightly varying during the measurement.

Figure 5b and Figure 6b show the measured temperature recorded by the fibers wound on the two bellows with increasing burning time. The measured temperature increases fast at the early stage and the temperature rise rate of the fiber decreases gradually with the time increase. As shown in Figure 5b and Figure 6b, the temperature recorded by the fiber at lengths 486–490 m wound on bellow I is displayed. The temperature recorded at a fiber length of 488 m is the highest which corresponds to the closest location to the fire source. The location of the fiber at 488 m length on bellow I corresponds to x = 50 cm, which means that the fire source is located at 50 cm away. In the same way, the temperature curve recorded for fiber lengths of 266 m and 267 m almost coincides with the temperatures measured at 265–269 m. This corresponds to a length of bellow II between 44.4 cm and 50 cm, *i.e.*, the fire is located between 44.4 cm and 50 cm. Combining the above experimental results, the abscissa of the fire source is obtained as 44.4–50 cm, *i.e.*, its width is 5.6 cm and the maximum temperature position of the fire source is close to 50 cm.

**Figure 7:**
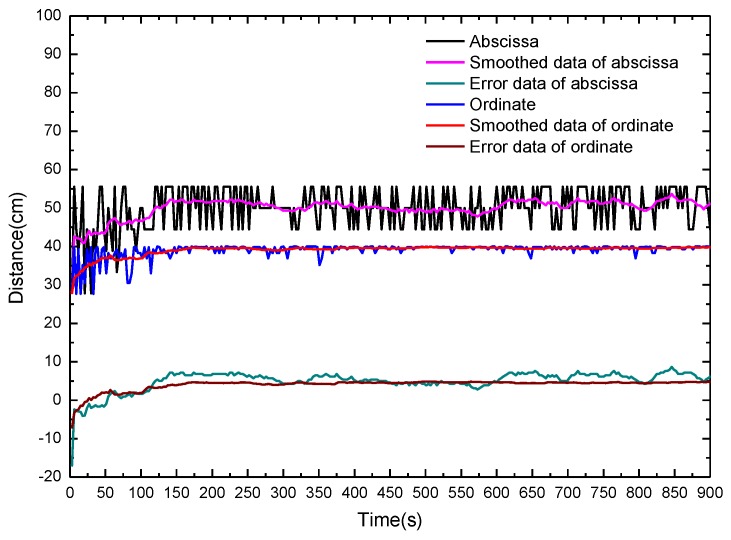
Calculated fire source location and location errors with fibers on opposite sides of fire source in dependence of the alcohol burning time.

Figure 7 shows the calculated fire source location and the location errors with fibers on opposite sides of the fire source in dependence of the alcohol burning time. With the proposed method and the measured temperature maximum on bellow I, the abscissa x and ordinate y of the fire source can be determined. Obviously, the calculated abscissa x uncertainties are rather large during the whole measurement time. The main reason related to this phenomenon is that the fire source cannot be seen as a point fire source and the surrounding environment turbulence leads to the observed fluctuation of the calculated results. A moving average filter is adopted to overcome this problem. The smoothed distance fluctuations are significantly reduced compared with the original data. The corresponding errors of abscissa and ordinate positions after smoothing the data are shown in Figure 7. The error ranges of the abscissa and ordinate of the fire source within the burning time 10 s to 60 s are (−4.09 cm, 2.04 cm) and (−2.60 cm, 2.09 cm), respectively. The position of the fire source is determined quickly with an acceptable error range.

**Figure 8:**
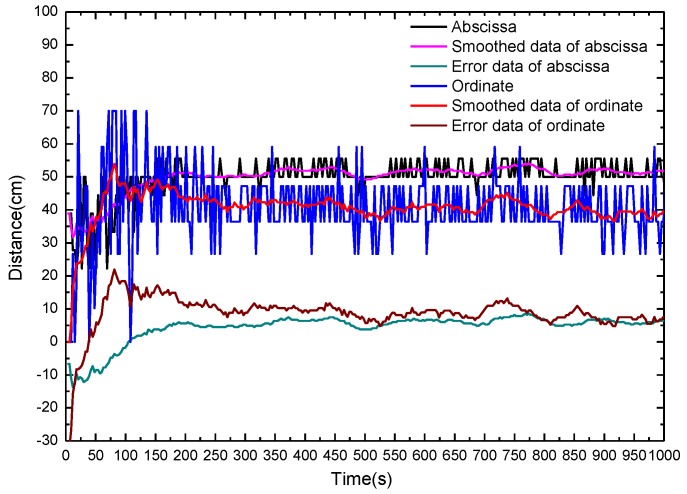
Calculated fire source location and location errors with fibers on same side of fire source in dependence of the alcohol burning time.

The calculated fire source location and the location errors with fibers on the same side of fire source in dependence of the alcohol burning time is shown in Figure 8. The calculated ordinate y fluctuates intensely at the beginning of the fire, and gradually stabilizes after 3 min. According to the experimental conditions, thresholds of abscissa and ordinate are set as (0, 100) and (0, 70), respectively. If the calculated values are beyond the scope of the threshold value, the threshold value of minimum and maximum are used instead of the values below or above the threshold, respectively. The fluctuations are reduced evidently after the data are smoothed.

For fire burning times longer than 3 min, the errors of the calculated data are stable, and the maximum errors of the abscissa and ordinate of the fire source are 8.57 cm and 12.43 cm, respectively. Fire source localization determination with fibers on the same side of the fire source takes longer than that with fibers on opposite sides because that the area and flame of burning alcohol is so small that the heat generated at the early stage of alcohol burning stage is small. In addition, the heat flow of transmission can be blocked partly by bellow I. Since the wavefront of the hot gases has no longer a regular circular shape, the calculated position data fluctuate intensely. The measurement errors can be significantly reduced by improving the method of fire source localization and the DTS system such as employing an appropriate data processing method, optimizing thermal capacity of the sensing fiber, and so on. A further possibility for improvement of the accuracy and precision could consist in inserting a third sensing fiber orthogonal to the other two but on the cost of complexity and at higher cost of the whole system.

**Figure 9:**
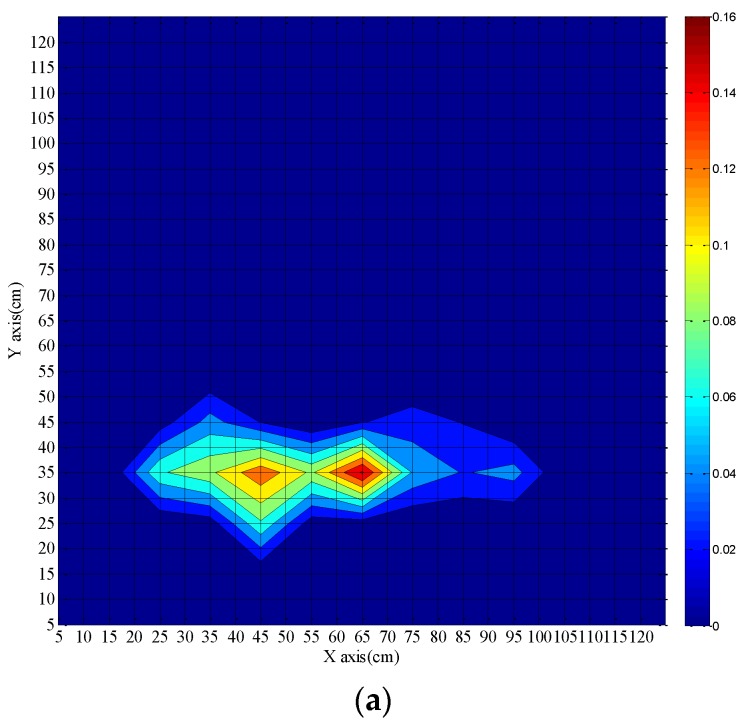

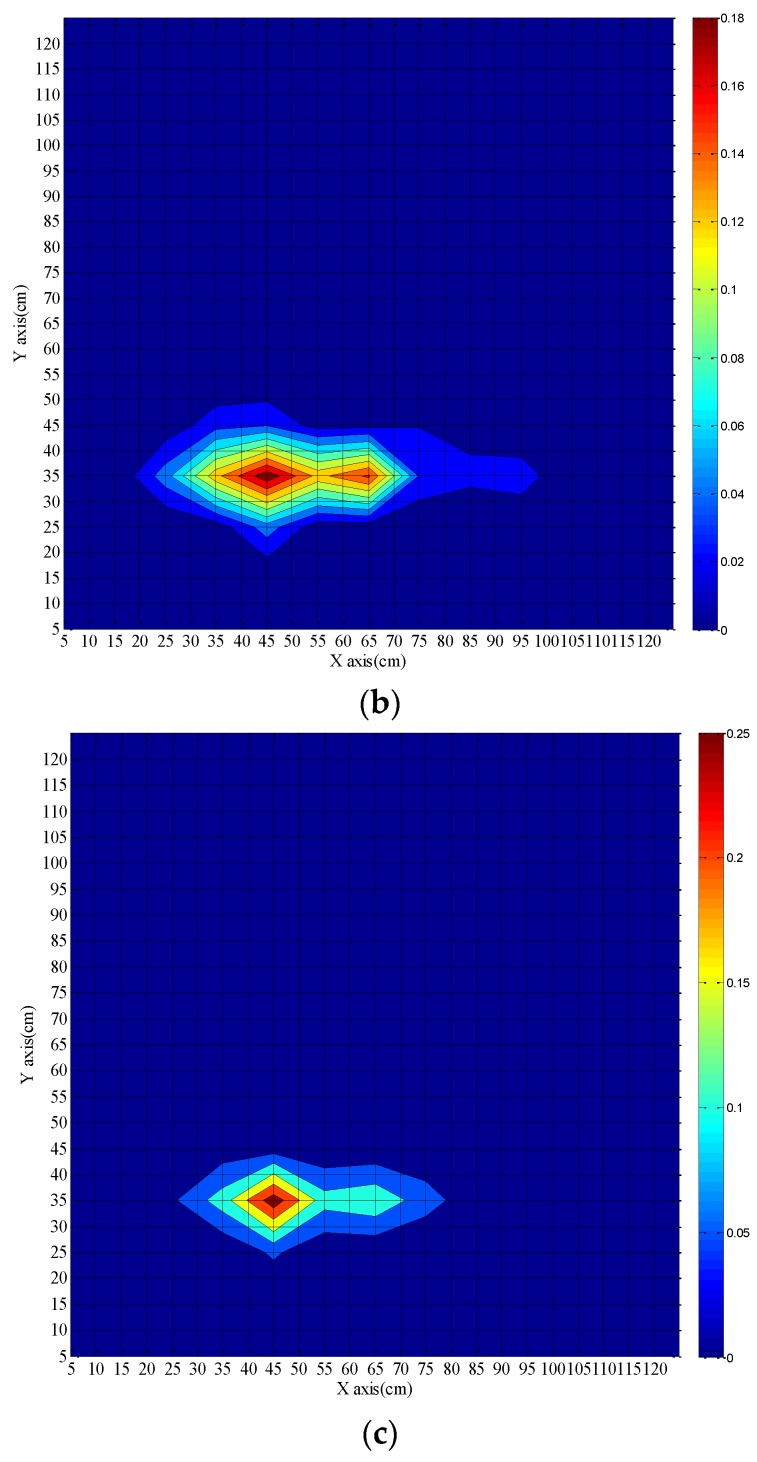
Calculated fire source location with fibers on opposite sides of fire source for different burning times of the fire. (**a**): 40 s; (**b**) 60 s; (**c**) 90 s.

**Figure 10:**
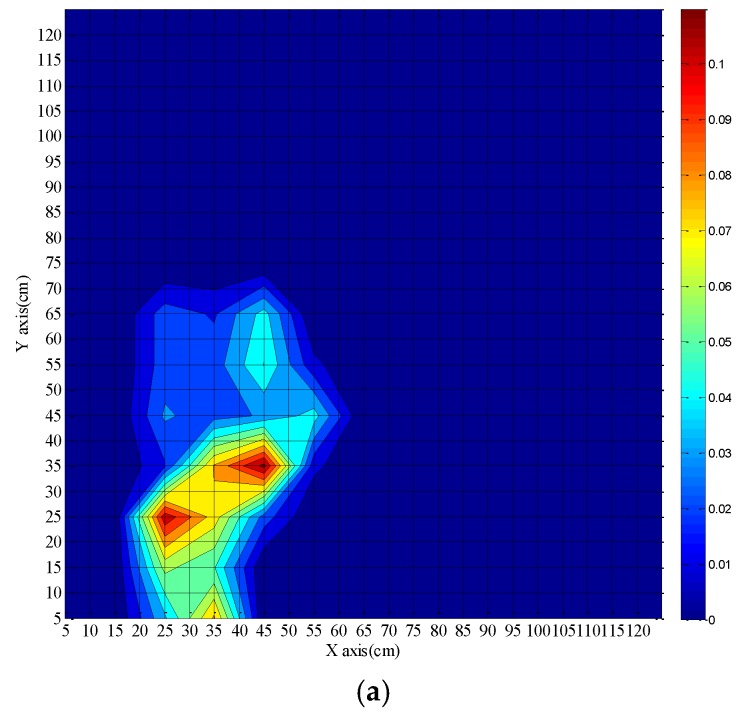

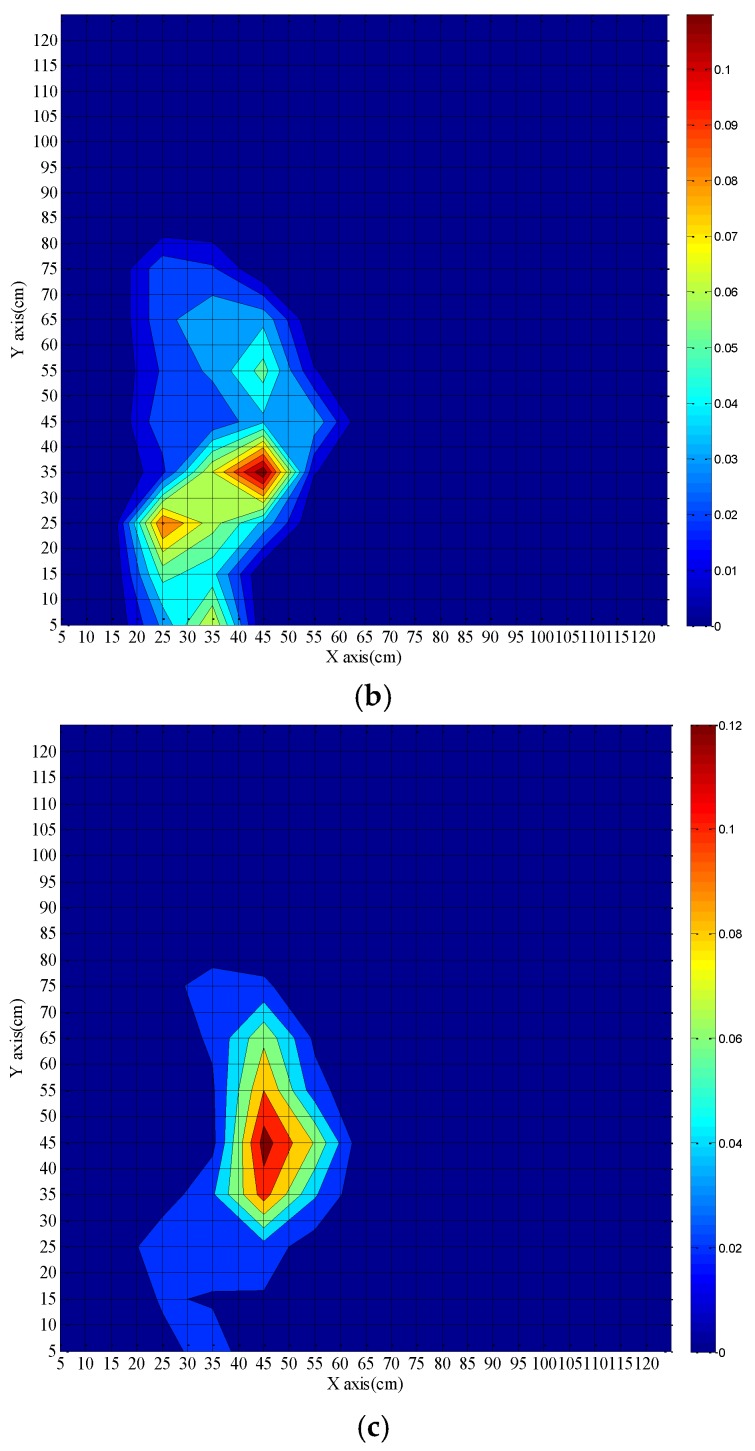
Calculated fire source location with fibers on same side of fire source for different burning times of the fire. (**a**): 70 s; (**b**) 80 s; (**c**) 180 s.

The ability of the DTS system for fire source localization has been discussed above. Due to the real-time nature of the DTS measurement, we get more coordinate data with increasing time. During the measurement, the coordinate data slightly vary around the real position of the fire source and some of them are overlapping. The possible area of the fire source (here is 1.2 m × 1.2 m) is partitioned with a grid (5 cm × 5 cm) spacing, and the coordinate data falling into each grid square are counted. The counting number finally corresponds to a probability. Hence the real fire source is located in the square grid with the highest probability. As a result the position of the fire source is determined. The results are depicted in Figure 9 and Figure 10. The color bar represents the probability of the calculated position of the fire source. The calculated coordinates of the fire source location are plotted for different times. Figure 9 shows results after 40 s, 60 s, and 90 s fire burning time when the fibers are placed on opposite sides of the fire source. The extent of the actual fire source is 11.0 cm × 8.0 cm and the center position is (45 cm, 35 cm). For a fire burning time of 40 s, the shape of the fire source looks irregular almost like two independent fire sources. The coordinates of the two fire centers are (45 cm, 35 cm) and (65 cm, 35 cm), and the coordinate of the most possible position is (65 cm, 35 cm). When the burning time is 60 s, the coordinate of the most possible position changes to (45 cm, 35 cm). The shape of the fire source becomes more regular. When the fire burning time is ≥90 s the coordinate of the fire source is determined at (45 cm, 35 cm). Although there are two centers of fire sources when the burning time is 40 s, the calculated fire source location can cover the actual fire source well and the fire source location becomes more accurate with increasing burning time. As a result, the fire source position can be calculated roughly for 40 s and the accurate position can be achieved for 90 s burning time, which is very helpful for extinguishing the fire.

Figure 10 shows results after 70 s, 80 s, and 180 s fire burning time when the fibers are placed on the same side of the fire source. The center position of the actual fire source is (45.5 cm, 32 cm). As mentioned above, the fluctuations of the calculated fire source location in Figure 7 are different from those in Figure 8, thus the shape and range of the fire source in Figure 9 are quite different from that in Figure 10.

The fire source location is preliminarily confirmed when the burning time is 70 s, and the actual fire source is covered completely within the calculated fire source. The position of the fire source is finally determined at a burning time of 180 s, which is consistent with the previous discussions. The time of 40 s for a rough determination of a fire source position is considerably shorter than the 90 s reported in [5] and the location error of the proposed method is much smaller than that achieved with sensor arrays [5,6,11].

## 5. Conclusions

We present a new method for localizing a fire source with a distributed temperature sensor (DTS) system. Two parallel optical fibers are used as the sensing elements. The theoretical analysis of the fire source localization is discussed and experimental results are presented. The results indicate that the spatial errors for the fire localization with the proposed method are acceptable and that the model can predict the position of the actual fire source well for burning times of more than 40 s. The advantages of the DTS system lie in its simplicity and efficiency. The scheme can also be applied in places with long-distances and wide ranges, such as highway, mine or subway tunnels.

## References

[1] Yu L., Wang N., Meng X. Real-time forest fire detection with wireless sensor networks. Proceedings of the 11th Annual International Conference on Networking and Mobile Computing.

[2] Chen T.-H., Wu P.-H., Chiou Y.-C. An early fire-detection method based on image processing. Proceedings of the 2004 International Conference on Image Processing.

[3] Xia D., Wang S., Zhu M., Tang H. A method research on fire source localization using dual-line gas sensor array. Preceedings of the 7th World Congress on Intelligent Control and Automation.

[4] Chen S.-J., Hovde D.C., Peterson K.A., Marshall A.W. (2007). Fire detection using smoke and gas sensors. Fire Saf. J..

[5] Kaiser T. Fire detection with temperature sensor arrays. Proceedings of the IEEE International Carnahan Conference on Security Technology.

[6] Berentsen M, Kaiser T. Fire Location Estimation Using Temperature Sensor Arrays. Proceedings 12th International Conference on Automatic Fire Detection.

[7] Richards R.F., Munk B.N., Plumb O.A. (1997). Fire detection, location and heat release rate through inverse problem solution. Part I: Theory. Fire Saf. J..

[8] Richards R.F., Munk B.N., Plumb O.A. (1997). Fire detection, location and heat release rate through inverse problem solution. Part II: Experiment. Fire Saf. J..

[9] Moreno J.M., Oechel W.C. (1989). A simple method for estimating fire intensity after a burn in California chaparral. Acta Ecol..

[10] Henig-Sever N., Poliakov D., Broza M. (2001). A novel method for estimation of wild fire intensity based on ash pH and soil microarthropod community. Pedobiologia.

[11] Wang S., Berentsen M., Kaiser T. (2005). Signal processing algorithms for fire localization using temperature sensor arrays. Fire Saf. J..

[12] Wang Z.L., Zhang S., Chang J., Liu Y.N. (2014). Adaptive data acquisition algorithm in Raman distributed temperature measurement system. Opt. Int. J. Light Electron Opt..

[13] Hausner M.B., Suárez F., Glander K.E., van de Giesen N., Selker J.S., Tyler S.W. (2011). Calibrating single-ended fiber-optic Raman spectra distributed temperature sensing data. Sensors.

[14] Dakin J.P., Pratt D.J., Bibby G.W., Ross J.N. Temperature distribution measurement using Raman ratio thermometry. Proceedings of the Fiber Optic and Laser Sensors III.

[15] Bolognini G., Hartog A. (2013). Raman-based fibre sensors: Trends and applications. Opt. Fiber Technol..

[16] Signorini A., Nannipieri T., Gabella L., Pasquale F.D., Latini G., Ripari D. Raman distributed temperature sensor for oil leakage detection in soil: A field trial and future trends. Proceedings of the 23rd International Conference on Optical Fibre Sensors.

[17] Vercauteren N., huwald H., Bou-Zeid E., Selker J.S., Lemmin U., Parlange M.B., Lunati I. (2011). Evolution of superficial lake water temperature profile under diurnal radiative forcing. Water Resour. Res..

[18] Yilmaz G., Karlik S.E. (2006). A distributed optical fiber sensor for temperature detection in power cables. Sens. Actuators A Phys..

[19] Scarcia W., Palma G., Falconi M.C., de Leonardis F., Passaro V.M.N., Prudenzano F. (2015). Electromagnetic Modelling of Fiber Sensors for Low-Cost and High Sensitivity Temperature Monitoring. Sensors.

[20] Jin L., Zhang W., Zhang H., Liu B., Zhao J., Tu Q., Kai G., Dong X. (2006). An embedded FBG sensor for simultaneous measurement of stress and temperature. IEEE Photonics Technol. Lett..

[21] Wang Z.L., Zhang S.S., Chang J., Lv G.P., Wang W.J., Jiang S., Liu X.Z., Liu X.H., Luo S., Sun B.N. (2013). Attenuation auto-correction method in Raman distributed temperature measurement system. Opt. Quantum Electron..

